# Harm and Addiction Perceptions of the JUUL E-Cigarette Among Adolescents

**DOI:** 10.1093/ntr/ntz183

**Published:** 2019-09-26

**Authors:** Christopher Russell, Evangelos Katsampouris, Neil Mckeganey

**Affiliations:** Centre for Substance Use Research, Glasgow, United Kingdom

## Abstract

**Introduction:**

This study assessed adolescents’ harm and addiction perceptions of the highest-selling brand—JUUL—of the most commonly used tobacco product—electronic cigarettes (e-cigarettes)—among adolescents in the United States.

**Methods:**

A cross-sectional online survey assessed use and perceptions of the harmfulness and addictiveness of the JUUL e-cigarette and conventional tobacco cigarettes in a nationally representative sample of 9865 adolescents aged 13–17 years in the United States. Associations between adolescents’ harm and addiction perceptions and their use of a JUUL e-cigarette were examined through multinomial logistic regression models.

**Results:**

Overall, 6.1% and 9.3% of adolescents believed daily use and occasional use of a JUUL e-cigarette, respectively, would cause them no harm. Around 11.3% believed they would either never experience harm from using a JUUL e-cigarette or they could use a JUUL e-cigarette for at least 20 years before experiencing any harm, and 7.3% believed they would be “very unlikely” to become addicted to using a JUUL e-cigarette. Overall, 39.3% and 29.3% of adolescents perceived the JUUL e-cigarette as “less harmful” and “less addictive” than conventional cigarettes, respectively. Compared to never users of the JUUL e-cigarette, current and former users held significantly lower harm and addiction perceptions of the JUUL e-cigarette on all measures.

**Conclusions:**

The majority of adolescents believed using a JUUL e-cigarette would put them at, at least, some risk for experiencing health problems and addiction. A smaller but significant proportion believed they could use a JUUL e-cigarette without ever being harmed by or becoming addicted to the JUUL e-cigarette.

**Implications:**

The study reports adolescents’ perceptions of the harmfulness and addictiveness of the highest-selling brand of the most commonly used tobacco product among youth in the United States. Though the majority of adolescents correctly believed that using a JUUL e-cigarette would put them at, at least, some risk for experiencing health problems and addiction, a small proportion believed that using a JUUL e-cigarette would be risk free. Correcting such risk-free perceptions may reduce adolescents’ interest in trying and continuing to use JUUL e-cigarettes.

## Introduction

Electronic cigarettes (“e-cigarettes”) are battery-powered devices that can deliver nicotine and flavorings to the user in the form of an aerosol by heating a liquid rather than by burning tobacco. E-cigarettes are the most commonly used tobacco product among middle and high school students in the United States, and have been so since 2014.^1^ Youth use of e-cigarettes surged considerably between 2017 and 2018, with around 5 out of 100 (4.9%) middle school students and 21 out of 100 (20.8%) high school students in 2018 now estimated to have used an e-cigarette in the past 30 days (compared to 3.3% and 11.7%, respectively, in 2017). These data indicate that around 3.5 million middle and high school students have used an e-cigarette in the past 30 days, up from around 2 million in 2017. In response to these data, the US Food and Drug Administration (FDA) announced in September 2018 that e-cigarette use among youth had become “an epidemic.” ^2^

Though e-cigarette aerosol typically contains fewer and lower concentrations of toxicants and carcinogens than are typically carried in smoke from combustible tobacco cigarettes, e-cigarette aerosol is not harmless, and regular, long-term inhalation is unlikely to be without biological effects in humans. E-cigarette use poses unique health and addiction risks during adolescence.^3,4^ E-cigarette aerosol can contain metals, organic volatile compounds, and flavoring additives that may cause respiratory harm when inhaled, particularly to adolescents.^3^ Using e-cigarettes has also been shown to increase adolescents’ risk of ever-smoking combustible tobacco cigarettes and increase adolescents’ frequency and intensity of subsequent combustible tobacco cigarette smoking.^3^ Understanding factors that may have driven the recent surge in youth use of e-cigarettes is critically important to public health efforts to prevent and reduce youth e-cigarette use and their known and as-yet-unknown associated health and addiction risks.

Harm and addiction perceptions provide a strong empirical basis for explaining why adolescents initiate, substitute, and continue to use e-cigarettes and other tobacco products. Adolescents typically perceive e-cigarettes to be equally or less harmful than conventional cigarettes,^5–15^ and adolescents who hold lower harm perceptions, lower addiction perceptions, and higher benefit perceptions of e-cigarettes are more likely to have used, start using, or continue to use e-cigarettes.^16–20^ In 2017, the proportions of 8th, 10^th^, and 12th graders in the United States who believed regular e-cigarette use poses a “great risk of harm” were 20%, 19%, and 16%, respectively.^21^ This pattern of a decreasing belief in the harmfulness of regular e-cigarette use with age during adolescence contrasts against an increasing belief in the harmfulness of regular cigarette smoking. These data suggest that the majority of adolescents are either unaware of the potential health risks of e-cigarette use or underestimate their likelihood of experiencing harm from using e-cigarettes, which may in turn have contributed to recent increases in adolescents’ willingness to experiment with and continue using e-cigarettes.

In late 2018, FDA launched “The Real Cost” Youth E-Cigarette Prevention Campaign to educate US youth aged 12–17 years about the harmful and potentially harmful effects of using e-cigarettes. The core message communicated by this campaign is that, just like smoking regular cigarettes, using e-cigarettes puts adolescents at risk for becoming addicted to nicotine and developing various health problems. The Real Cost campaign puts particular focus on the dangers of “pod-based e-cigarettes,” a particular class of e-cigarettes that resemble USB flash drives in size, weight, and appearance, which have become increasingly popular among youth. The growth in popularity of one brand of pod-based e-cigarette—JUUL—has been singled out by the FDA as a particular cause for concern.

The JUUL e-cigarette is a tech-inspired vaping device that, at 9.45 × 1.50 × 0.69 cm and weighing 100 g, is small enough to fit in a closed fist. The JUUL e-cigarette is based on a two-part system: a prefilled, disposable e-liquid pod that clicks into a small battery. All 0.7-mL e-liquid pods marketed by JUUL in the United States are designed to contain either 23 mg of nicotine (3% nicotine by weight) or 40 mg of nicotine (5% nicotine by weight). Despite a lack of data on the safety of JUUL vaping products, 2018 market tracking data showed that past 52-week retail sales of JUUL products in the United States increased from $150.0 million in July 2017 (+652.9% vs. July 2016)^22^ to $1.3 billion in August 2018 (+761.4% vs. August 2017),^23^ making JUUL the first e-cigarette brand to record over $1 billion in sales in a 52-week period through tracked channels. Contemporaneous with this growth in sales, however, has been an alarming increase in the frequency of media and anecdotal reports from parents, educators, school superintendents, health care providers, and public health experts who have claimed the use of JUUL e-cigarettes had become widespread among middle and high school students both within and outwith school premises,^24–32^ with around 6% of 15–17-year olds estimated to have used a JUUL e-cigarette in the past 30 days.^33^ To the extent that the JUUL e-cigarette has become uniquely popular among US youth, the possibility that the JUUL brand of vaping products may be having a larger effect on US adolescent health compared to other brands and styles of e-cigarette provides a strong rationale for research that seeks to quantify and explain youth use of JUUL vaping products specifically.

Little is known about how adolescents perceive their risk of being harmed by or becoming addicted to using a JUUL e-cigarette or the role played by JUUL risk perceptions in adolescents’ use decisions. This study aimed to provide first estimates of adolescents’ harm and addiction perceptions of the JUUL e-cigarette and conventional cigarettes and, specifically, to estimate the proportions of adolescents who believe that using a JUUL e-cigarette and smoking conventional cigarettes would pose no risk to their health.

## Methods

### Participants

Participants were adolescents aged 13–17 years in the United States who were children of adults enrolled as panelists of a Qualtrics’ Internet research panel and who had heard of or seen a brand of e-cigarette called “JUUL.” Qualtrics’ Internet research panel comprises a diverse sample of over 30 million adults in the United States who have volunteered to periodically receive invitations to complete surveys online in exchange for incentives. Panelists consent/give assent to each survey they decide to participate in and are free to withdraw from any survey at any time.

Participants were recruited to the study in two ways. First, panelists who were identified by Qualtrics as having at least one child aged 13–17 years living in the household were sent an invitation by e-mail. Second, a notification of this survey opportunity was posted to online portals to which Qualtrics panelists have access. It was not possible to know how many panelists saw the study invitation posted in the online portals or how many e-mail invitations were received or read. To avoid self-selection bias, neither the survey invitation nor the portal notification included specific details about the survey contents or topics.

Recruitment quotas were set with the intention of constructing a nonprobability sample that matched the US adolescent population in terms of age, gender, and US census region. Additionally, to correct for survey nonresponse and possible selection bias, a study-specific poststratification weight was used to adjust the composition of the final sample to match the age, gender, and regional distributions of US adolescent population. Demographic and geographic distributions from the March 2017 supplement of the US Census Bureau’s Current Population Survey (CPS) were employed as population benchmarks for sample recruitment and adjustment and included gender (male and female), age (13, 14, 15, 16, and 17), and census region (Northeast, Midwest, South, and West).

Surveys were completed online between November 23 and December 13, 2018. A total of 28 850 panelists started the survey, of whom 3035 (10.5%) did not meet basic eligibility criteria (1839 did not have children; 1114 did not have a child aged 13–17 years living in the household; 82 did not live in the United States), 7094 (24.6%) did not give consent/assent to participate, and 6554 (22.7%) children screened out due to being unaware of e-cigarettes (*n* = 974) or unaware of a brand of e-cigarette called “JUUL” (*n* = 5580). Of the 12 167 eligible children, 1136 (3.9% of invited) were screened out due to quota restrictions; 1159 (4.0% of invited) were excluded due to low-quality or incomplete responses; and 8 (0.0% of invited) were excluded for failing to report their age.

This left a final analytic sample of 9865 US adolescents who were aware of e-cigarettes and aware of the JUUL brand of e-cigarettes. Demographic, cigarette smoking status and JUUL use status characteristics (unweighted *n*; weighted %) of the analytic sample are summarized in Supplementary Table A. Participants were predominantly male (51.0%), aged 15–17 years (60.5%), non-Hispanic White (68.6%), and living in the South (37.2%); 13.2% were current cigarette smokers, 24.7% were former smokers, and 62.0% were never-smokers. With regard to use of a JUUL e-cigarette, 15.7% were current users, 11.5% were former users, and 72.7% were never users.

### Procedure

Clicking the Web-link in the e-mail invitation/portal notification routed the panelist to an online Parent Permission Form (PPF). This PPF explained that *Qualtrics* was seeking the panelist’s permission to invite their child to take part in an online survey about their child’s views and experiences of tobacco products, such as cigarettes and e-cigarettes. The PPF provided information about the purpose of the study, who was conducting the study, what their child’s participation would involve, what their child would receive for participating, their child’s rights as a study participant—including their right to skip questions or withdraw at any time—how their child’s information would be protected, the contact details of the study director, assurances of participant anonymity and confidentiality, and contact details for the *Qualtrics* support center and of the Institutional Review Board that was providing oversight of this study. Panelists were asked to allow their child to complete the survey and submit their answers in private.

When a panelist gave consent for their child to participate, the panelist was routed to an online Youth Assent Form (YAF), which they were asked to read and then ask their child to read carefully before deciding whether he/she wished to participate. The YPF provided the same information and assurances as the PPF. The survey took around 25 minutes to complete. Upon completion, a message displayed thanking participants for their time and informing them that a credit equivalent to $10 would be deposited to their parent’s panel account and that their parent has been asked to give $10 to the participant. This study was approved by the Advarra Institutional Review Board (approval no. 00030080, October 2, 2018).

### Measures

#### Demographics

Questions assessed participants’ age, gender, ethnicity, race, school grade, and state of residence.

#### Tobacco Product Use

Ever-smoking was assessed by the question, “Have you ever tried cigarette smoking, even one or two puffs?” Participants who responded “No” to this question were defined as “never-smokers.” Participants who responded “Yes” to this question were defined as ever-smokers and subsequently asked, “When was the last time you smoked a cigarette, even one or two puffs? (Please choose the first answer that fits).” Those who responded “Earlier today,” “Not today but sometime during the past 7 days,” or “Not during the past 7 days but sometime during the past 30 days” were defined as “current smokers.” Those who responded “Not during the past 30 days but sometime during the past 6 months,” “Not during the past 6 months but sometime during the past year,” “1 to 4 years ago,” or “5 or more years ago” were defined as “former smokers.” Participants’ status as a current, former, or never user of a JUUL e-cigarette was determined by the same questions and response options, with “used a JUUL e-cigarette” substituted for “tried cigarette smoking/smoked a cigarette” in each question.

#### Absolute Harm Perceptions

Absolute harm perceptions were assessed by four questions: “How much do you think people harm themselves when they: (i) use a JUUL e-cigarette on some days; (ii) use a JUUL e-cigarette every day; (iii) smoke cigarettes on some days; (iv) smoke cigarettes every day?” (no harm; a little harm; some harm; a lot of harm).

Perceptions of the length of time for which a person would have to use a JUUL e-cigarette before experiencing harm were assessed by the question, “How long do you think someone has to use a JUUL e-cigarette before it harms their health?” (it will never harm their health; <1 year; 1 year; 5 years; ≥20 and more years; don’t know). Perceptions of the length of time for which a person would have to smoke cigarettes before experiencing harm was assessed by the same question and response options, with “smoke cigarettes” substituted for “use a JUUL e-cigarette.”

#### Absolute Addiction Perceptions

Addiction perceptions of the JUUL e-cigarette were assessed by the question, “How likely is someone to become addicted to using a JUUL e-cigarette?” (very unlikely; somewhat unlikely; neither likely nor unlikely; somewhat likely; very likely). Perceived likelihood of becoming addicted to cigarettes was assessed by the same question, with “smoking cigarettes” substituted for “using a JUUL e-cigarette.”

#### Relative Harm and Addiction Perceptions

Perceptions of the relative harmfulness of using a JUUL e-cigarette and smoking conventional cigarettes were assessed by the question, “Do you believe using a JUUL e-cigarette is less harmful, about the same, or more harmful than smoking regular cigarettes?” (less harmful; equally harmful; more harmful; I don’t know enough about these products). Perceptions of the relative addictiveness of using a JUUL e-cigarette and smoking conventional cigarettes were assessed by the same question and response options, with “addictive” substituted for “harmful.”

### Data Quality Checks

Several manual and automated checks were implemented to ensure participants who gave low-quality or invalid responses were excluded from the analytic sample. Checks were conducted for straight-lining, geolocation, inattentiveness, speeding, duplicates, and bots.

### Data Analysis

Population-weighted proportions and 95% confidence intervals are reported for all perception measures, stratified by age group, gender, cigarette smoking status, and JUUL use status. Associations between adolescents’ harm and addiction perceptions of using a JUUL e-cigarette and their cigarette smoking status (current smoker vs. former smoker vs. never-smoker) and JUUL use status (current user vs. former user vs. never user), adjusted for the effects of age and gender, were examined through multinomial logistic regression models. The effects of current and former use of cigarettes and the effects of current and former use of a JUUL e-cigarette on adolescents’ harm and addiction perceptions of the JUUL e-cigarette were tested by specifying “never-smokers” and “never JUUL users” as the reference groups in all models. The response option that indicated the highest perception of harm or addiction for each question was specified as the reference option, with the exception of two models in which “equally harmful” and “equally addictive” were specified as the respective reference options. *p* values ≤0.05 were considered statistically significant.

## Results

### Harm Perceptions

#### Daily Use of a JUUL E-Cigarette

Overall, 6.1% and 45.9% of adolescents believed using a JUUL e-cigarette every day would cause “no harm” and “a lot harm,” respectively. A belief that daily use of a JUUL e-cigarette would cause “no harm” was reported by 14.5% of current JUUL users, 10.1% of former users, and 3.6% of never users (Table 1). Compared to never JUUL users, current and former users were 6.3 times (adjusted odds ratio (aOR) = 6.32; 4.86, 8.23) and 3.6 times (aOR = 3.64; 2.80, 4.80) more likely, respectively, to believe that daily use of a JUUL e-cigarette would cause “no harm” (compared to “a lot of harm”; Supplementary Table B). Compared to never-smokers, current and former smokers were 3.2 times (aOR = 3.22; 2.41, 4.30) and 2.1 times (aOR = 2.10; 1.63, 2.63) more likely, respectively, to believe that daily use of a JUUL e-cigarette would cause “no harm” (compared to “a lot of harm”).

**Table 1.** Harm perceptions of daily use of a JUUL e-cigarette and conventional cigarettes

Predictor variable	JUUL e-cigarette				Conventional cigarettes			
	No harm	A little harm	Some harm	A lot of harm	No harm	A little harm	Some harm	A lot of harm
	% (95% CI)	% (95% CI)	% (95% CI)	% (95% CI)	% (95% CI)	% (95% CI)	% (95% CI)	% (95% CI)
Total	6.1 (5.6, 6.5)	18.4 (17.7, 19.2)	29.5 (28.6, 30.4)	45.9 (45.0, 46.9)	1.0 (0.8, 1.1)	4.1 (3.7, 4.5)	16.7 (16.0, 17.5)	78.2 (77.4, 79.0)
Sex								
Male	6.6 (5.9, 7.3)	18.9 (17.8, 20.0)	30.4 (29.1, 31.7)	44.1 (42.8, 45.5)	1.1 (0.8, 1.4)	4.4 (3.8, 4.9)	17.8 (16.8, 18.9)	76.7 (75.5, 77.8)
Female	5.5 (4.9, 6.1)	17.9 (16.8, 18.9)	28.6 (27.3, 29.8)	48.1 (46.6, 49.5)	0.8 (0.6, 1.1)	3.8 (3.2, 4.3)	15.5 (14.5, 16.6)	79.9 (78.7, 81.0)
Age group								
13–14	6.0 (5.2, 6.7)	17.1 (15.9, 18.3)	28.9 (27.5, 30.3)	48.0 (46.5, 49.6)	1.1 (0.8, 1.4)	4.1 (3.5, 4.7)	16.3 (15.1, 17.5)	78.5 (77.2, 79.8)
15–17	6.1 (5.5, 6.7)	19.3 (18.3, 20.3)	29.9 (28.8, 31.1)	44.7 (43.4, 45.9)	0.9 (0.6, 1.1)	4.1 (3.6, 4.6)	17.0 (16.1, 18.0)	78.0 (77.0, 79.1)
Cigarette smoking status								
Current smoker	13.0 (11.2, 14.9)	32.5 (29.9, 35.0)	33.8 (31.3, 36.4)	20.7 (18.5, 22.9)	3.1 (2.2, 4.1)	12.9 (11.1, 14.7)	34.6 (32.1, 37.2)	49.3 (46.6, 52.0)
Former smoker	8.5 (7.4, 9.6)	25.7 (24.0, 27.5)	33.5 (31.6, 35.4)	32.3 (30.4, 34.1)	0.7 (0.4, 1.1)	4.6 (3.8, 5.4)	23.0 (21.4, 24.7)	71.6 (69.8, 73.4)
Never-smoker	3.6 (3.2, 4.1)	12.5 (11.7, 13.4)	27.0 (25.9, 28.1)	56.8 (55.6, 58.1)	0.6 (0.4, 0.8)	2.0 (1.7, 2.4)	10.4 (9.7, 11.2)	86.9 (86.1, 87.8)
JUUL use status								
Current JUUL user	14.5 (12.7, 16.3)	34.0 (31.6, 36.3)	33.1 (30.8, 35.5)	18.4 (16.5, 20.3)	2.1 (1.4, 2.9)	9.8 (8.3, 11.3)	30.9 (28.6, 33.2)	57.1 (54.7, 59.6)
Former JUUL user	10.1 (8.3, 11.8)	28.3 (25.7, 30.9)	34.6 (31.8, 37.3)	27.1 (24.5, 29.7)	1.9 (1.1, 2.7)	6.0 (4.6, 7.4)	25.6 (23.1, 28.2)	66.5 (63.7, 69.2)
Never JUUL user	3.6 (3.2, 4.0)	13.5 (12.7, 14.3)	27.9 (26.9, 29.0)	54.9 (53.8, 56.1)	0.5 (0.4, 0.7)	2.5 (2.2, 2.9)	12.3 (11.5, 13.1)	84.6 (83.7, 85.4)

Questions were: “How much do you think people harm themselves when they (a) use a JUUL e-cigarette every day; and (b) smoke cigarettes every day?”

CI = confidence interval.

#### Occasional Use of a JUUL E-Cigarette

Overall, 9.3% and 28.9% of adolescents believed using a JUUL e-cigarette on some days but not every day would cause “no harm” and “a lot harm,” respectively. A belief that occasional use of a JUUL e-cigarette would cause “no harm” was reported by 20.2% of current JUUL users, 16.2% of former users, and 5.8% of never users (Supplementary Table C). Compared to never JUUL users, current and former users were 5.5 times (aOR = 5.49; 4.30, 7.10) and 3.8 times (aOR = 3.83; 3.00, 4.94) more likely, respectively, to believe that occasional use of a JUUL e-cigarette would cause “no harm” (compared to “a lot of harm”; Supplementary Table D). Compared to never smokers, current and former smokers were 2.8 times (aOR = 2.76; 2.10, 3.62) and 2.4 times (aOR = 2.39; 1.94, 2.94) more likely, respectively, to believe that occasional use of a JUUL e-cigarette would cause “no harm” (compared to “a lot of harm”).

#### Perceived Length of Time Needed to Use a JUUL E-Cigarette Before Experiencing Harm

A total of 11.3% of adolescents believed they would either never experience any harm from using a JUUL e-cigarette or that they could use a JUUL e-cigarette for at least 20 years before experiencing harm. Approximately, 18.6% of current JUUL users, 24.7% of former users, and 40.6% of never users reported a belief that people would experience harm with “less than 1 year” of use of a JUUL e-cigarette (Table 2). Compared to never JUUL users, current and former users were 4.4 times (aOR = 4.39; 3.35, 5.75) and 2.8 times (aOR = 2.75; 2.07, 3.65) more likely, respectively, to believe that people would never harm their health by using a JUUL e-cigarette (compared to “less than 1 year” to experience harm; Supplementary Table E). Compared to never-smokers, current and former smokers were 2.5 times (aOR = 2.53; 1.87, 3.41) and 2.4 times (aOR = 2.35; 1.84, 2.99) more likely, respectively, to believe that people would “never” harm their health by using a JUUL e-cigarette (compared to “less than 1 year”).

**Table 2.** Perceived length of time for which a person has to use a JUUL e-cigarette and smoke cigarettes before experiencing harm

Variable	JUUL e-cigarette						
	Will never harm health	<1 year	1 year	5 years	10 years	≥20 years	Don’t know
	% (95% CI)	% (95% CI)	% (95% CI)	% (95% CI)	% (95% CI)	% (95% CI)	% (95% CI)
Total	5.8 (5.3, 6.2)	35.3 (34.3, 36.2)	15.9 (15.2, 16.7)	14.0 (13.3, 14.6)	6.9 (6.4, 7.4)	5.5 (5.1, 6.0)	16.6 (15.8, 17.3)
Sex							
Male	6.5 (5.8, 7.2)	34.3 (33.0, 35.6)	16.3 (15.3, 17.3)	14.4 (13.4, 15.3)	7.2 (6.5, 7.9)	5.7 (5.0, 6.3)	15.7 (14.7, 16.7)
Female	5.0 (4.4, 5.6)	36.5 (35.1, 37.8)	15.6 (14.6, 16.6)	13.6 (12.6, 14.6)	6.5 (5.8, 7.2)	5.3 (4.7, 6.0)	17.5 (16.5, 18.6)
Age group							
13–14	5.6 (4.9, 6.4)	35.5 (34.0, 37.0)	15.9 (14.7, 17.0)	14.8 (13.7, 15.9)	6.0 (5.3, 6.7)	5.2 (4.5, 5.9)	17.1 (15.9, 18.2)
15–17	5.9 (5.3, 6.5)	35.2 (34.0, 36.4)	16.0 (15.1, 16.9)	13.4 (12.6, 14.3)	7.5 (6.8, 8.1)	5.7 (5.1, 6.3)	16.3 (15.3, 17.2)
Cigarette smoking status							
Current smoker	11.5 (9.7, 13.2)	21.9 (19.7, 24.2)	16.4 (14.4, 18.4)	17.5 (15.5, 19.6)	10.2 (8.6, 11.9)	9.3 (7.7, 10.9)	13.2 (11.3, 15.0)
Former smoker	8.8 (7.6, 9.9)	26.5 (24.8, 28.3)	15.2 (13.7, 16.6)	16.3 (14.8, 17.8)	9.1 (8.0, 10.3)	7.5 (6.5, 8.6)	16.6 (15.1, 18.1)
Never-smoker	3.4 (3.0, 3.9)	41.6 (40.4, 42.9)	16.2 (15.2, 17.1)	12.3 (11.5, 13.1)	5.3 (4.7, 5.8)	3.9 (3.4, 4.4)	17.3 (16.4, 18.3)
JUUL use status							
Current user	12.9 (11.3, 14.6)	18.6 (16.6, 20.5)	15.4 (13.6, 17.3)	17.6 (15.7, 19.5)	10.6 (9.1, 12.1)	10.1 (8.6, 11.7)	14.7 (13.0, 16.5)
Former user	9.7 (8.0, 11.4)	24.7 (22.2, 27.2)	15.4 (13.3, 17.5)	17.1 (14.9, 19.3)	9.3 (7.6, 11.0)	8.6 (7.0, 10.3)	15.2 (13.1, 17.2)
Never user	3.6 (3.2, 4.0)	40.6 (39.5, 41.7)	16.1 (15.3, 17.0)	12.7 (11.9, 13.5)	5.7 (5.2, 6.2)	4.0 (3.6, 4.5)	17.2 (16.3, 18.1)
Variable	Conventional cigarettes						
	Will never harm health	<1 year	1 year	5 years	10 years	≥20 years	Don’t know
	% (95% CI)	% (95% CI)	% (95% CI)	% (95% CI)	% (95% CI)	% (95% CI)	% (95% CI)
Total	0.8 (0.6, 0.9)	41.6 (40.6, 42.5)	15.6 (14.9, 16.4)	16.0 (15.3, 16.7)	8.5 (8.0, 9.1)	6.5 (6.0, 7.0)	11.0 (10.3, 11.6)
Sex							
Male	0.8 (0.6, 1.1)	40.3 (39.0, 41.7)	15.9 (14.9, 16.9)	16.9 (15.9, 18.0)	8.8 (8.0, 9.6)	7.0 (6.3, 7.7)	10.1 (9.3, 10.9)
Female	0.7 (0.5, 0.9)	42.9 (41.5, 44.3)	15.4 (14.3, 16.4)	15.0 (14.0, 16.0)	8.2 (7.5, 9.0)	6.0 (5.3, 6.7)	11.8 (10.9, 12.7)
Age group							
13–14	0.8 (0.5, 1.0)	41.2 (39.6, 42.7)	16.2 (15.1, 17.4)	16.2 (15.0, 17.3)	8.1 (7.2, 8.9)	6.2 (5.5, 7.0)	11.4 (10.4, 12.4)
15–17	0.8 (0.5, 1.0)	41.8 (40.6, 43.1)	15.2 (14.3, 16.2)	15.9 (15.0, 16.8)	8.9 (8.1, 9.6)	6.7 (6.1, 7.4)	10.7 (9.9, 11.5)
Cigarette smoking status							
Current smoker	1.6 (0.9, 2.3)	22.5 (20.2, 24.8)	18.0 (15.9, 20.1)	22.1 (19.9, 24.4)	13.4 (11.5, 15.2)	13.4 (11.6, 15.3)	9.0 (7.4, 10.5)
Former smoker	0.9 (0.5, 1.2)	34.9 (33.1, 36.8)	15.4 (13.9, 16.8)	19.2 (17.6, 20.8)	10.8 (9.6, 12.1)	8.8 (7.7, 9.9)	10.0 (8.8, 11.2)
Never-smoker	0.5 (0.4, 0.7)	48.3 (47.0, 49.5)	15.2 (14.3, 16.1)	13.4 (12.6, 14.3)	6.6 (6.0, 7.2)	4.1 (3.7, 4.6)	11.8 (11.0, 12.6)
JUUL use status							
Current user	1.2 (0.6, 1.7)	25.4 (23.2, 27.5)	17.8 (15.9, 19.7)	21.8 (19.7, 23.9)	13.6 (11.9, 15.4)	12.3 (10.7, 13.9)	8.0 (6.6, 9.3)
Former user	0.9 (0.3, 1.4)	30.6 (27.9, 33.3)	15.7 (13.6, 17.8)	21.3 (18.9, 23.7)	11.1 (9.3, 12.9)	11.5 (9.7, 13.4)	8.9 (7.2, 10.6)
Never user	0.6 (0.5, 0.8)	46.8 (45.6, 47.9)	15.2 (14.3, 16.0)	13.9 (13.1, 14.7)	7.0 (6.4, 7.6)	4.5 (4.0, 5.0)	11.9 (11.2, 12.7)

The question was: “How long do you think someone has to use a JUUL e-cigarette/smoke cigarettes before it harms their health?”

CI = confidence interval.

#### Perceived Likelihood of Becoming Addicted to Using a JUUL E-Cigarette

Overall, 7.3% and 35.3% of adolescents believed they would be “very unlikely” and “very likely,” respectively, to become addicted to using a JUUL e-cigarette. Approximately 11.1% of current JUUL users, 8.8% of former users, and 6.2% of never users believed they would be “very unlikely” to become addicted to using a JUUL e-cigarette (Table 3). Compared to never JUUL users, current and former users were 2.6 times (aOR = 2.58; 2.00, 3.35) and 2.2 times (aOR = 2.15; 1.63, 2.84) more likely, respectively, to believe they would be “very unlikely” to become addicted to using a JUUL e-cigarette (compared to “very likely”; (Supplementary Table F). Compared to never-smokers, current and former smokers were approximately 71% (aOR = 1.71; 1.29, 2.28) and 81% (aOR = 1.81; 1.46, 2.26) more likely, respectively, to believe they would be “very unlikely” to become addicted to using a JUUL e-cigarette (compared to “very likely”).

**Table 3.** Perceived likelihood of becoming addicted to using a JUUL e-cigarette and smoking combustible cigarettes

Predictor variable	JUUL e-cigarette				
	Very unlikely	Somewhat unlikely	Neither likely nor unlikely	Somewhat likely	Very likely
	% (95% CI)	% (95% CI)	% (95% CI)	% (95% CI)	% (95% CI)
Total	7.3 (6.8, 7.8)	10.0 (9.4, 10.6)	12.5 (11.9, 13.2)	34.8 (33.9, 35.7)	35.3 (34.3, 36.2)
Sex					
Male	7.4 (6.7, 8.1)	10.1 (9.3, 11.0)	13.1 (12.1, 14.0)	34.8 (33.4, 36.1)	34.7 (33.3, 36.0)
Female	7.1 (6.4, 7.8)	9.8 (9.0, 10.6)	12.0 (11.1, 12.9)	35.0 (33.7, 36.4)	36.1 (34.7, 37.4)
Age group					
13–14	7.8 (7.0, 8.6)	10.0 (9.1, 11.0)	11.7 (10.7, 12.7)	34.4 (32.9, 35.9)	36.1 (34.6, 37.6)
15–17	7.0 (6.3, 7.6)	10.0 (9.2, 10.7)	13.1 (12.3, 14.0)	35.1 (33.9, 36.3)	34.8 (33.6, 36.0)
Cigarette smoking status					
Current smoker	9.9 (8.3, 11.6)	15.9 (14.0, 17.9)	19.3 (17.2, 21.5)	32.5 (30.0, 35.1)	22.3 (20.0, 24.5)
Former smoker	8.7 (7.6, 9.8)	15.0 (13.6, 16.4)	15.8 (14.4, 17.3)	36.6 (34.7, 38.6)	23.9 (22.2, 25.6)
Never smoker	6.2 (5.6, 6.8)	6.8 (6.1, 7.4)	9.8 (9.1, 10.6)	34.6 (33.4, 35.8)	42.6 (41.4, 43.9)
JUUL use status					
Current JUUL user	11.1 (9.5, 12.6)	16.7 (14.8, 18.5)	19.1 (17.1, 21.0)	33.2 (30.9, 35.6)	19.9 (17.9, 21.9)
Former JUUL user	8.8 (7.2, 10.5)	17.5 (15.3, 19.7)	16.9 (14.7, 19.1)	36.5 (33.7, 39.3)	20.3 (18.0, 22.7)
Never JUUL user	6.2 (5.6, 6.8)	7.4 (6.8, 8.0)	10.5 (9.8, 11.2)	34.9 (33.8, 36.0)	41.0 (39.9, 42.1)
Predictor variable	Conventional cigarettes				
	Very unlikely	Somewhat unlikely	Neither likely nor unlikely	Somewhat likely	Very likely
	% (95% CI)	% (95% CI)	% (95% CI)	% (95% CI)	% (95% CI)
Total	4.2 (3.8, 4.6)	3.7 (3.3, 4.1)	5.8 (5.3, 6.2)	28.6 (27.7, 29.5)	57.7 (56.7, 58.7)
Sex					
Male	4.3 (3.8, 4.9)	3.9 (3.4, 4.4)	6.3 (5.7, 7.0)	29.8 (28.5, 31.0)	55.6 (54.2, 57.0)
Female	4.1 (3.5, 4.7)	3.5 (3.0, 4.0)	5.2 (4.6, 5.8)	27.3 (26.0, 28.5)	59.9 (58.6, 61.3)
Age group					
13–14	4.7 (4.0, 5.3)	4.4 (3.8, 5.1)	5.9 (5.2, 6.7)	27.2 (25.9, 28.6)	57.7 (56.2, 59.3)
15–17	3.9 (3.4, 4.4)	3.2 (2.8, 3.7)	5.7 (5.1, 6.3)	29.5 (28.3, 30.6)	57.7 (56.4, 58.9)
Cigarette smoking status					
Current smoker	5.2 (4.0, 6.4)	8.4 (6.9, 9.9)	10.8 (9.1, 12.5)	34.6 (32.1, 37.2)	40.9 (38.2, 43.5)
Former smoker	4.4 (3.6, 5.2)	4.9 (4.0, 5.7)	6.3 (5.3, 7.3)	34.4 (32.6, 36.3)	49.9 (48.0, 51.9)
Never-smoker	3.9 (3.5, 4.4)	2.3 (1.9, 2.6)	4.5 (4.0, 5.0)	24.9 (23.9, 26.0)	64.4 (63.2, 65.6)
JUUL use status					
Current JUUL user	5.1 (4.0, 6.2)	7.8 (6.5, 9.2)	8.4 (7.0, 9.8)	34.2 (31.9, 36.6)	44.4 (42.0, 46.9)
Former JUUL user	5.0 (3.8, 6.3)	5.7 (4.4, 7.1)	7.8 (6.3, 9.4)	34.8 (32.0, 37.6)	46.6 (43.7, 49.5)
Never JUUL user	3.9 (3.4, 4.3)	2.5 (2.1, 2.9)	4.9 (4.4, 5.4)	26.4 (25.4, 27.4)	62.3 (61.1, 63.4)

The question was: “How likely is someone to become addicted to using a JUUL e-cigarette/smoking cigarettes?”

### Relative Harm Perceptions

Overall, 39.3% of adolescents believed using a JUUL e-cigarette is “less harmful” than smoking conventional cigarettes; 39.2% believed using a JUUL e-cigarette is “equally harmful” as smoking cigarettes. Using a JUUL e-cigarette was perceived as “less harmful” than smoking cigarettes by 60.4% of current JUUL users, 59.6% of former users, and 31.6% of never users (Table 4). Compared to never JUUL users, current and former users were 3.2 times (aOR = 3.20; 2.73, 3.76) and 2.5 times (aOR = 2.52; 2.16, 2.95) more likely, respectively, to believe using a JUUL e-cigarette is “less harmful” than smoking cigarettes (compared to “equally harmful”; Supplementary Table G). Compared to never-smokers, former smokers were approximately 47% (aOR = 1.47; 1.30, 1.65) more likely to believe that using a JUUL e-cigarette is “less harmful” than smoking cigarettes (compared to “equally harmful”).

**Table 4.** Relative harm and addiction perceptions of using a JUUL e-cigarette and smoking conventional cigarettes

Predictor variable	Perceived harmfulness of JUUL vs. cigarettes*				Perceived addictiveness of JUUL vs. cigarettes**			
	Less harmful	Equally harmful	More harmful	Don’t know	Less addictive	Equally addictive	More addictive	Don’t know
	% (95% CI)	% (95% CI)	% (95% CI)	% (95% CI)	% (95% CI)	% (95% CI)	% (95% CI)	% (95% CI)
Total	39.3 (38.4, 40.3)	39.2 (38.2, 40.1)	11.8 (11.1, 12.4)	9.7 (9.1, 10.3)	29.3 (28.4, 30.2)	51.6 (50.6, 52.6)	11.0 (10.4, 11.7)	8.0 (7.5, 8.5)
Sex								
Male	40.2 (38.9, 41.6)	38.5 (37.1, 39.8)	12.1 (11.2, 13.0)	9.2 (8.4, 10.0)	30.2 (29.0, 31.5)	50.6 (49.3, 52.1)	11.3 (9.5, 11.2)	7.8 (7.2, 8.7)
Female	38.4 (37.0, 39.7)	40.0 (38.6, 41.3)	11.4 (10.6, 12.3)	10.2 (9.4, 11.1)	28.3 (27.1, 29.6)	52.6 (51.2, 54.0)	10.8 (9.9, 11.6)	8.3 (7.5, 9.1)
Age group								
13–14	37.7 (36.2, 39.3)	39.2 (37.7, 40.8)	12.5 (11.4, 13.5)	10.5 (9.6, 11.5)	27.5 (26.1, 28.9)	51.4 (49.9, 53.0)	12.1 (11.0, 13.1)	8.9 (8.1, 9.8)
15–17	40.4 (39.1, 41.6)	39.2 (37.9, 40.4)	11.3 (10.5, 12.1)	9.1 (8.4, 9.9)	30.4 (29.3, 31.6)	51.7 (50.5, 53.0)	10.4 (9.6, 11.2)	7.4 (6.7, 8.1)
Cigarette smoking status								
Current smoker	49.6 (46.9, 52.3)	30.8 (28.3, 33.3)	16.1 (14.1, 18.1)	3.5 (2.5, 4.5)	40.8 (38.1, 43.4)	42.5 (39.9, 45.3)	13.6 (11.8, 15.5)	3.0 (2.1, 3.9)
Former smoker	52.5 (50.5, 54.5)	31.8 (30.0, 33.7)	9.8 (8.6, 11.0)	5.9 (4.9, 6.8)	42.8 (40.8, 44.7)	43.4 (41.5, 45.4)	8.2 (8.3, 7.2)	5.5 (4.6, 6.4)
Never-smoker	31.9 (30.8, 33.1)	44.0 (42.7, 45.2)	11.6 (10.8, 12.4)	12.5 (11.7, 13.3)	21.5 (20.5, 22.5)	56.8 (55.6, 58.1)	11.6 (11.6, 10.8)	10.1 (9.3, 10.8)
JUUL use status								
Current JUUL user	60.4 (57.9, 62.8)	23.9 (21.8, 26.1)	13.0 (11.3, 14.7)	2.7 (1.9, 3.5)	49.8 (47.3, 52.3)	37.0 (34.6, 39.4)	11.0 (9.4, 12.5)	2.2 (1.5, 2.9)
Former JUUL user	59.6 (56.8, 62.5)	28.1 (25.5, 30.7)	8.5 (6.9, 10.2)	3.7 (2.6, 4.8)	46.7 (43.8, 49.6)	41.2 (38.3, 44.1)	7.8 (6.2, 9.3)	4.3 (3.1, 5.5)
Never JUUL user	31.6 (30.5, 32.7)	44.2 (43.1, 45.4)	12.0 (11.3, 12.8)	12.1 (11.4, 12.9)	22.1 (21.2, 23.1)	56.4 (55.3, 57.6)	11.6 (10.8, 12.3)	9.9 (9.2, 10.6)

*The question was: “Do you believe using a JUUL e-cigarette is less harmful, about the same, or more harmful than smoking cigarettes?”

**The question was: “Do you believe using a JUUL e-cigarette is less addictive, about the same, or more addictive than smoking cigarettes?”

### Relative Addiction Perceptions

Overall, 29.3% of adolescents believed using a JUUL e-cigarette is “less addictive” than smoking conventional cigarettes; 51.6% believed using a JUUL e-cigarette would be “equally addictive” as smoking cigarettes. Using a JUUL e-cigarette was perceived as “less addictive” than smoking cigarettes by 49.8% of current JUUL users, 46.7% of former users, and 22.1% of never users (Table 4). Compared to never JUUL users, current and former users were 2.7 times (aOR = 2.72; 2.34, 3.15) and 2.2 times (aOR = 2.21; 1.90, 2.60) more likely, respectively, to believe that using a JUUL e-cigarette is “less addictive” than smoking cigarettes, respectively (compared to “equally addictive”) (Supplementary Table H). Compared to never smokers, current and former smokers were approximately 33% (aOR = 1.33; 1.13, 1.57) and 75% (aOR = 1.75; 1.55, 1.98) more likely, respectively, to believe that using a JUUL e-cigarette is “less addictive” than smoking cigarettes (compared to “equally addictive”).

## Discussion

The study assessed adolescents’ perceptions of the harmfulness and addictiveness of the highest selling brand of the most commonly used tobacco product among youth in the United States. The majority of adolescents believed not only that using a JUUL e-cigarette would pose fewer risks to health than smoking conventional cigarettes but also that using a JUUL e-cigarette would carry at least some risk of developing health problems and addiction. A smaller but significant proportion of adolescents, however, held the erroneous belief that using a JUUL e-cigarette would be risk free, even if used for many years, and unlikely to ever lead to addiction. Adolescents who were currently using a JUUL were most likely to perceive the JUUL as posing low to no risk to their health. Previous research on tobacco harm perceptions would predict that adolescents who hold such low and zero-risk harm and addiction perceptions may be more open to start or continue using a JUUL e-cigarette or switching from other tobacco products to a JUUL e-cigarette either because they are unaware of the potential health and addiction risks of using a JUUL e-cigarette use or because they underestimate their likelihood of eventually personally experiencing harm from using a JUUL e-cigarette.

These data are important given that the health and addiction risks of long-term use of the JUUL e-cigarette, both in an absolute sense and relative to the use of other tobacco products, are not yet known and likely will not be well characterized for several years. In the absence of data on the human health impact of using JUUL e-cigarettes, and communication of these data in forms that are comprehensible to adolescents, risk perceptions of the JUUL e-cigarette provide adolescents with a strong basis for making decisions as to whether to start, stop, switch to, or continue using a JUUL e-cigarette.

Present findings suggest that public health efforts to discourage youth uptake and continuation of JUUL e-cigarette use may benefit from educational campaigns and interventions that help adolescents to more fully appreciate that, though using a JUUL e-cigarette *may* not be as harmful as smoking regular cigarettes, the JUUL e-cigarette is harmful to adolescents’ health, whether or not they have ever or currently smoke cigarettes. These interventions should ideally specify the harms that adolescents may experience through different use frequencies, intensities, and durations and address the misperception, held by a minority of adolescents in this study, that, because a person has not yet experienced health problems and addiction from using a JUUL e-cigarette, that person is not now accumulating harm through continued use that may manifest in the future as a serious health problem or addiction.

The concepts of “safer ≠ safe” and “not harmful now ≠ not harmful forever” may be usefully explained to adolescents by conveying what the best available data currently show—all tobacco products carry risks to health, but different tobacco products carry different risks to people at different ages when used over different periods of time. Reframing adolescents’ understanding of tobacco products as uniformly risky to one of different tobacco and nicotine products occupying different levels on a continuum of risk may more effectively increase the salience of the harms that a nonsmoker may experience by starting to use e-cigarettes over and above the salience of the harms that a nonsmoker would avoid by starting to use e-cigarettes instead of regular cigarettes. Additionally, positioning e-cigarettes on a risk continuum relative to conventional tobacco cigarettes may more effectively communicate to adolescents that so extreme are the health risks posed by smoking cigarettes that other tobacco products can be much less harmful than cigarettes while still having potential to cause a great deal of harm.

While this study provides important first estimates of the prevalence of low harm and addiction perceptions of the JUUL e-cigarette among adolescents, the findings must be interpreted in the context of several limitations. First, though the study sample was constructed to be representative of US adolescents in terms of age, gender, and region, the generalizability of results to US adolescents may be limited as the study sample was recruited from online research panels and because approximately 42.2% of invited, consenting, and otherwise eligible adolescents were excluded as they had not seen or heard of a brand of e-cigarette called “JUUL” before taking part in this study. A number of studies have shown, however, that the application of corrections (e.g. quota-based recruitment and population weighting) to nonprobability samples is effective in producing prevalence estimates that match those estimated from probability samples.^34,35^ Additionally, the corrections applied in this study were specific to the US adolescent population, and so results are unlikely to represent youth perceptions of the JUUL e-cigarette in other countries.

Second, current and former JUUL users in this study were significantly more likely than never JUUL users to hold low-risk perceptions of using a JUUL e-cigarette. This association is consistent with previous research on the association between risk perceptions and adolescents’ use of e-cigarettes more broadly.^16–20^ However, it is important to stress that the cross-sectional nature of this survey prevents conclusions about whether low-risk perceptions among current JUUL users were factors that motivated these adolescents to try using a JUUL e-cigarette or whether low-risk perceptions were consequences of having used a JUUL e-cigarette for some time. Perceiving the JUUL e-cigarette and e-cigarettes more broadly as zero or low risk may have been one of several factors that contributed to the surge in prevalence of past 30-day e-cigarette use among youth between 2017 and 2018 and of particular concern to e-cigarette use among adolescents who had never previously smoked a cigarette. However, it is equally possible that adolescents’ risk perceptions of the JUUL e-cigarette decreased over a period of use in which few harms or symptoms of addiction were experienced. Longitudinal study designs are needed to assess the prospective relationship between preuse risk perceptions and subsequent use of a JUUL e-cigarette and change in preuse risk perceptions following periods of experimental and regular use of the JUUL e-cigarette. Third, and relatedly, the risk perceptions of the JUUL e-cigarette observed in the present study, and of e-cigarette use more broadly, are subject to change as new research emerges, media reporting increases, social attitudes change, and new regulations and legislation are implemented.

Last, this study did not seek to identify the sources of information that contributed to participants’ perceptions of and decisions to use the JUUL e-cigarette. Future assessments of youth perceptions of e-cigarette harms should identify the sources that adolescents are most likely to seek, use, and trust for information and opinions on e-cigarette use and to identify the sources of misinformation on e-cigarettes that most commonly foster adolescents’ misperceptions of e-cigarettes as more or less harmful than the available science suggests them to be would be an important future focus of research.

In conclusion, this study found that while most adolescents aged 13–17 years believed using a JUUL e-cigarette would carry at least some risk of developing health problems and addiction, a small but significant proportion of adolescents believed using a JUUL e-cigarette would cause them no harm or risk of becoming addicted. Continued surveillance of adolescents’ changing harm and addiction perceptions of the JUUL e-cigarette, and the role played by these perceptions in adolescents’ decisions to try and continue using the JUUL e-cigarette, can inform the development of public health messages that differentiate the relative and absolute harms of using a JUUL e-cigarette during adolescence.

## Supplementary Material

ntz183_suppl_Supplementary_Material
