# Supplementary material for: Harm and Addiction Perceptions of the JUUL E-Cigarette Among Adolescents
Source: Nicotine Tob Res. 2019 Sep 26;22(5):713–21. doi: 10.1093/ntr/ntz183 (PMC7171269; doi:10.1093/ntr/ntz183)
Supplement: ntz183_suppl_Supplementary_Material [file ntz183_suppl_supplementary_material.docx]

**Supplementary Material**

Supplementary Table A. Demographic characteristics, cigarette smoking status and JUUL use status of U.S. adolescents.

| Variable | Unweighted n (Weighted %) |
| --- | --- |
| Total | 9,865 (100.0) |
| **Demographic Variables** |  |
| Sex |  |
| Male | 4,963 (51.0) |
| Female | 4,891 (48.9) |
| Missing | 11 (0.1) |
| Age Group |  |
| 13-14 | 3,872 (39.5) |
| 15-17 | 5,993 (60.5) |
| Race/Ethnicity |  |
| Non-Hispanic, White | 6,922 (68.6) |
| Non-Hispanic, Black | 860 (8.4) |
| Non-Hispanic, American Indian/Alaskan | 99 (1.1) |
| Non-Hispanic, Asian, Hawaiian or PI§ | 144 (1.7) |
| Non-Hispanic, 2 or More Races | 480 (4.9) |
| Hispanic† | 1,305 (14.7) |
| Missing | 55 (0.6) |
| School Grade |  |
| Middle School | 2,515 (25.8) |
| High School | 7,256 (73.2) |
| Missing | 94 (1.0) |
| U.S. Census Region |  |
| Midwest | 2,558 (21.3) |
| South | 4,119 (37.2) |
| West | 1,437 (23.4) |
| Northeast | 1,739 (18.0) |
| Missing | 12 (0.1) |
| **Smoking and JUUL e-cigarette variables** |  |
| Cigarette Smoking Status |  |
| Current Smoker | 1,283 (13.2) |
| Former Smoker | 2,437 (24.7) |
| Never Smoker | 6,141 (62.0) |
| Missing | 4 (0.0) |
| JUUL Use Status |  |
| Current JUUL User | 1,538 (15.7) |
| Former JUUL User | 1,112 (11.5) |
| Never JUUL User | 7,207 (72.7) |
| Missing | 8 (0.1) |

**Abbreviations**: PI = Pacific Islander.

§ Includes Asian Indian, Chinese, Filipino, Japanese, Korean, Vietnamese, Guamanian, Chamorro, and Samoan.

†Includes Mexican, Cuban, Puerto Rican and ‘other Hispanic’ ethnicity.

Supplementary Table B. Multinomial logistic regression model information for factors associated with harm perceptions of daily use of the JUUL e-cigarette.

|  | No Harm | | A Little Harm | | Some Harm | |
| --- | --- | --- | --- | --- | --- | --- |
| Predictor Variable | Unadjusted OR (95% CI) | aOR  (95% CI) | Unadjusted OR (95% CI) | aOR  (95% CI) | Unadjusted OR (95% CI) | aOR  (95% CI) |
| Sex |  |  |  |  |  |  |
| Male | 1.30 (1.01-1.54)** | 1.19 (1.00-1.42) | 1.15 (1.03-1.30)* | 1.07 (0.96-1.20) | 1.16 (1.06-1.27)** | 1.11 (1.01-1.22)* |
| Female | Ref. | Ref. | Ref. | Ref. | Ref. | Ref. |
| Age Group |  |  |  |  |  |  |
| 13-14 | 0.91 (0.80-1.10) | 1.14 (0.95-1.36) | 0.82 (0.74-0.92)** | 0.99 (0.88-1.11) | 0.90 (0.82-1.00)* | 0.99 (0.91-1.10) |
| 15-17 | Ref. | Ref. | Ref. | Ref. | Ref. | Ref. |
| Smoking Status |  |  |  |  |  |  |
| Current Smoker | 9.82 (7.76-12.43)*** | 3.22 (2.41-4.30)*** | 7.11 (6.00-8.45)*** | 3.20 (2.62-3.92)*** | 3.50 (3.00-4.10)*** | 2.15 (1.78-2.59)*** |
| Former Smoker | 4.10 (3.33-5.02)*** | 2.10 (1.63-2.63)*** | 3.61 (3.20-4.20)*** | 2.24 (1.97-2.59)*** | 2.18 (2.00-2.50)*** | 1.70 (1.46-1.88)*** |
| Never Smoker | Ref. | Ref. | Ref. | Ref. | Ref. | Ref. |
| JUUL Use Status |  |  |  |  |  |  |
| Current JUUL User | 12.03 (9.69-14.92)*** | 6.32 (4.86-8.23)*** | 7.52 (6.40-8.83)*** | 3.89 (3.23-4.70)*** | 3.60 (3.04-4.14)*** | 2.32 (1.94-2.80)*** |
| Former JUUL User | 5.68 (4.43-7.30)*** | 3.64 (2.80-4.80)*** | 4.24 (3.60-5.04)*** | 2.64 (2.19-3.20)*** | 2.51 (2.15-3.00)*** | 1.85 (1.56-2.20)*** |
| Never JUUL User | Ref. | Ref. | Ref. | Ref. | Ref. | Ref. |

*** p < 0.001; ** p < 0.010; * p < 0.050; The reference category for the dependent variable was: ‘a lot of harm’.

**Note**: The question was: *“How much do you think people harm themselves when they use a JUUL e-cigarette every day?”*.

**Abbreviations**: aOR = adjusted odds ratio. Unadjusted ORs were estimated using only the relevant variable as the predictor variable.

Supplementary Table C. Harm perceptions of occasional use of a JUUL e-cigarette and conventional cigarettes.

|  | JUUL E-Cigarette | | | | Conventional Cigarettes | | | |
| --- | --- | --- | --- | --- | --- | --- | --- | --- |
|  | No Harm | A Little Harm | Some Harm | A Lot of Harm | No Harm | A Little Harm | Some Harm | A Lot of Harm |
| Predictor Variable | % (95% CI) | % (95% CI) | % (95% CI) | % (95% CI) | % (95% CI) | % (95% CI) | % (95% CI) | % (95% CI) |
| Total | 9.3 (8.7, 9.8) | 25.1 (24.3, 26.0) | 36.6 (35.6, 37.5) | 28.9 (28.0, 29.8) | 1.7 (1.4, 1.9) | 10.8 (10.2, 11.4) | 39.6 (38.7, 40.6) | 47.9 (46.9, 48.8) |
| Sex |  |  |  |  |  |  |  |  |
| Male | 9.9 (9.0, 10.7) | 25.9 (24.7, 27.1) | 36.3 (35.0, 37.7) | 27.9 (26.7, 29.2) | 1.9 (1.5, 2.3) | 11.3 (10.4, 12.1) | 40.1 (38.8, 41.5) | 46.6 (45.2, 47.9) |
| Female | 8.6 (7.8, 9.4) | 24.3 (23.1, 25.6) | 36.9 (35.6, 38.3) | 30.1 (28.8, 31.4) | 1.4 (1.1, 1.8) | 10.3 (9.4, 11.1) | 39.1 (37.7, 40.5) | 49.2 (47.8, 50.6) |
| Age Group |  |  |  |  |  |  |  |  |
| 13-14 | 8.9 (8.0, 9.8) | 24.0 (22.6, 25.3) | 37.0 (35.5, 38.5) | 30.2 (28.7, 31.6) | 2.0 (1.6, 2.5) | 10.3 (9.3, 11.2) | 38.3 (36.8, 39.8) | 49.4 (47.8, 51.0) |
| 15-17 | 9.5 (8.8, 10.3) | 25.9 (24.8, 27.0) | 36.4 (35.2, 37.6) | 28.2 (27.0, 29.3) | 1.5 (1.2, 1.8) | 11.1 (10.3, 11.9) | 40.5 (39.2, 41.7) | 46.8 (45.6, 48.1) |
| Cigarette Smoking Status |  |  |  |  |  |  |  |  |
| Current Smoker | 17.6 (15.5, 19.6) | 36.4 (33.8, 39.1) | 32.1 (29.6, 34.7) | 13.9 (12.0, 15.7) | 5.1 (3.9, 6.3) | 24.0 (21.7, 26.4) | 42.3 (39.6, 45.0) | 28.4 (26.0, 30.9) |
| Former Smoker | 13.7 (12.3, 15.1) | 32.8 (31.0, 34.7) | 34.6 (32.7, 36.5) | 18.8 (17.3, 20.4) | 1.8 (1.3, 2.4) | 15.4 (13.9, 16.8) | 44.8 (42.9, 46.8) | 37.9 (36.0, 39.9) |
| Never Smoker | 5.7 (5.2, 6.3) | 19.7 (18.7, 20.7) | 38.4 (37.2, 39.6) | 36.2 (35.0, 37.4) | 0.9 (0.7, 1.2) | 6.1 (5.5, 6.7) | 37.0 (35.8, 38.2) | 55.9 (54.7, 57.2) |
| JUUL Use Status |  |  |  |  |  |  |  |  |
| Current JUUL User | 20.2 (18.2, 22.2) | 39.0 (36.6, 41.5) | 28.9 (26.7, 31.2) | 11.8 (10.2, 13.5) | 3.6 (2.6, 4.5) | 21.1 (19.1, 23.2) | 43.4 (41.0, 45.9) | 31.9 (29.5, 34.2) |
| Former JUUL User | 16.2 (14.0, 18.3) | 36.7 (33.9, 39.5) | 31.6 (28.9, 34.3) | 15.5 (13.4, 17.7) | 3.2 (2.2, 4.2) | 16.9 (14.7, 19.1) | 45.8 (42.9, 48.7) | 34.2 (31.4, 36.9) |
| Never JUUL User | 5.8 (5.3, 6.4) | 20.3 (19.4, 21.3) | 39.1 (37.9, 40.2) | 34.8 (33.6, 35.9) | 1.0 (0.8, 1.3) | 7.6 (7.0, 8.2) | 37.8 (36.7, 38.9) | 53.5 (52.4, 54.7) |

**Note**: Questions were: *“How much do you think people harm themselves when they (a) use a JUUL e-cigarette on some days but not every day; and (b) smoke cigarettes on some days but not every day?”*

Supplementary Table D. Multinomial logistic regression model information for factors associated with harm perceptions of occasional use of the JUUL e-cigarette.

|  | JUUL No Harm | | A Little Harm | | Some Harm | |
| --- | --- | --- | --- | --- | --- | --- |
| Predictor Variable | Unadjusted OR (95% CI) | aOR  (95% CI) | Unadjusted OR (95% CI) | aOR  (95% CI) | Unadjusted OR (95% CI) | aOR  (95% CI) |
| Sex |  |  |  |  |  |  |
| Male | 1.23 (1.06-1.43)** | 1.13 (1.00-1.32) | 1.15 (1.03-1.30)* | 1.08 (0.96-1.20) | 1.06 (0.96-1.17) | 1.03 (0.93-1.13) |
| Female | Ref. | Ref. | Ref. | Ref. | Ref. | Ref. |
| Age Group |  |  |  |  |  |  |
| 13-14 | 0.87 (0.75-1.01) | 1.08 (0.92-1.27) | 0.86 (0.77-0.96)** | 1.02 (0.91-1.14) | 0.95 (0.90-1.05) | 1.01 (0.92-1.12) |
| 15-17 | Ref. | Ref. | Ref. | Ref. | Ref. | Ref. |
| Smoking Status |  |  |  |  |  |  |
| Current Smoker | 8.00 (6.40-10.00)*** | 2.76 (2.10-3.62)*** | 4.81 (4.00-5.80)*** | 2.30 (1.85-2.85)*** | 2.20 (1.81-2.62)*** | 1.70 (1.37-2.10)*** |
| Former Smoker | 4.60 (3.90-5.50)*** | 2.39 (1.94-2.94)*** | 3.20 (2.80-3.66)*** | 2.10 (1.77-2.40)*** | 1.73 (1.53-2.00)*** | 1.51 (1.31-1.74)*** |
| Never Smoker | Ref. | Ref. | Ref. | Ref. | Ref. | Ref. |
| JUUL Use Status |  |  |  |  |  |  |
| Current JUUL User | 10.20 (8.30-12.60)*** | 5.49 (4.30-7.10)*** | 5.64 (4.72-6.73)*** | 3.40 (2.80-4.20)*** | 2.17 (1.82-2.60)*** | 1.60 (1.30-2.00)*** |
| Former JUUL User | 6.20 (4.92-7.81)*** | 3.83 (3.00-4.94)*** | 4.02 (3.33-4.90)*** | 3.00 (2.20-3.30)*** | 1.81 (1.50-2.18)*** | 1.43 (1.20-1.74)** |
| Never JUUL User | Ref. | Ref. | Ref. | Ref. | Ref. | Ref. |

*** p < 0.001; ** p < 0.010; * p < 0.050; The reference category for the dependent variable was: ‘a lot of harm’.

**Note**: The question was: *“How much do you think people harm themselves when they use a JUUL e-cigarette on some days but not every day?”*.

**Abbreviations**: aOR = adjusted odds ratio. Unadjusted ORs were estimated using only the relevant variable as the predictor variable.

Supplementary Table E. Multinomial logistic regression model information for factors associated with perceptions of the length of time a person would need to use the JUUL e-cigarette before experiencing harm.

|  | Never | | 1 Year | | 5 Years | |
| --- | --- | --- | --- | --- | --- | --- |
| Predictor Variable | Unadjusted OR (95% CI) | aOR  (95% CI) | Unadjusted OR (95% CI) | aOR  (95% CI) | Unadjusted OR (95% CI) | aOR  (95% CI) |
| Sex |  |  |  |  |  |  |
| Male | 1.38 (1.15-1.65)*** | 1.28 (1.06-1.54)** | 1.12 (0.99-1.26) | 1.09 (0.96-1.23) | 1.13 (0.99-1.28) | 1.08 (0.95-1.22) |
| Female | Ref. | Ref. | Ref. | Ref. | Ref. | Ref. |
| Age Group |  |  |  |  |  |  |
| 13-14 | 0.96 (0.80-1.15) | 1.19 (0.98-1.43) | 0.99 (0.87-1.11) | 1.04 (0.92-1.17) | 1.09 (0.96-1.24) | 1.21 (1.07-1.38)** |
| 15-17 | Ref. | Ref. | Ref. | Ref. | Ref. | Ref. |
| Smoking Status |  |  |  |  |  |  |
| Current Smoker | 6.37 (5.00-8.13)*** | 2.53 (1.87-3.41)*** | 1.93 (1.59-2.34)*** | 1.43 (1.14-1.80)** | 2.72 (2.24-3.29)*** | 1.76 (1.40-2.21)*** |
| Former Smoker | 4.01 (3.25-4.95)*** | 2.35 (1.84-2.99)*** | 1.47 (1.27-1.70)*** | 1.26 (1.07-1.50)** | 2.08 (1.80-2.42)*** | 1.66 (1.40-2.00)*** |
| Never Smoker | Ref. | Ref. | Ref. | Ref. | Ref. | Ref. |
| JUUL Use Status |  |  |  |  |  |  |
| Current JUUL User | 7.81 (6.26-9.74)*** | 4.39 (3.35-5.75)*** | 2.10 (1.75-2.52)*** | 1.70 (1.36-2.11)*** | 3.03 (2.52-3.63)*** | 2.15 (1.73-2.66)*** |
| Former JUUL User | 4.41 (3.42-5.69)*** | 2.75 (2.07-3.65)*** | 1.57 (1.29-1.92)*** | 1.37 (1.10-1.70)** | 2.21 (1.81-2.69)*** | 1.70 (1.36-2.09)*** |
| Never JUUL User | Ref. | Ref. | Ref. | Ref. | Ref. | Ref. |

Supplementary Table E. (continued)

|  | 10 Years | | ≥ 20 Years | | Don’t Know | |
| --- | --- | --- | --- | --- | --- | --- |
| Predictor Variable | Unadjusted OR (95% CI) | aOR  (95% CI) | Unadjusted OR (95% CI) | aOR  (95% CI) | Unadjusted OR (95% CI) | aOR  (95% CI) |
| Sex |  |  |  |  |  |  |
| Male | 1.18 (1.00-1.39) | 1.12 (0.95-1.34) | 1.13 (0.95-1.36) | 1.07 (0.90-1.28) | 0.95 (0.85-1.07) | 0.94 (0.83-1.05) |
| Female | Ref. | Ref. | Ref. | Ref. | Ref. | Ref. |
| Age Group |  |  |  |  |  |  |
| 13-14 | 0.80 (0.67-0.95)* | 0.92 (0.77-1.10) | 0.90 (0.74-1.08) | 1.06 (0.88-1.28) | 1.04 (0.92-1.17) | 1.10 (0.98-1.24) |
| 15-17 | Ref. | Ref. | Ref. | Ref. | Ref. | Ref. |
| Smoking Status |  |  |  |  |  |  |
| Current Smoker | 3.68 (2.91-4.66)*** | 2.03 (1.53-2.71)*** | 4.52 (3.52-5.81)*** | 2.08 (1.53-2.83)*** | 1.45 (1.18-1.77)*** | 1.08 (0.86-1.37) |
| Former Smoker | 2.70 (2.23-3.27)*** | 1.95 (1.57-2.42)*** | 3.03 (2.45-3.74)*** | 1.91 (1.50-2.43)*** | 1.50 (1.30-1.73)*** | 1.32 (1.12-1.55)** |
| Never Smoker | Ref. | Ref. | Ref. | Ref. | Ref. | Ref. |
| JUUL Use Status |  |  |  |  |  |  |
| Current JUUL User | 4.07 (3.27-5.06)*** | 2.60 (1.97-3.34)*** | 5.52 (4.40-6.94)*** | 3.51 (2.65-4.65)*** | 1.87 (1.55-2.25)*** | 1.72 (1.38-2.13)*** |
| Former JUUL User | 2.68 (2.10-3.43)*** | 1.81 (1.40-2.37)*** | 3.51 (2.71-4.56)*** | 2.44 (1.83-3.26)*** | 1.44 (1.18-1.76)*** | 1.28 (1.03-1.60)* |
| Never JUUL User | Ref. | Ref. | Ref. | Ref. | Ref. | Ref. |

*** p < 0.001; ** p < 0.010; * p < 0.050; The reference category for the dependent variable was: ‘less than one year’.

**Note**: The question was: *“How long do you think someone has to use a JUUL e-cigarette before it harms their health?”*.

**Abbreviations**: aOR = adjusted odds ratio. Unadjusted ORs were estimated using only the relevant variable as the predictor variable.

Supplementary Table F. Multinomial logistic regression model information for factors associated with perceptions of the likelihood of becoming addicted to using a JUUL e-cigarette.

|  | Very Unlikely | | Somewhat Unlikely | |
| --- | --- | --- | --- | --- |
| Predictor Variable | Unadjusted OR (95% CI) | aOR  (95% CI) | Unadjusted OR (95% CI) | aOR  (95% CI) |
| Sex |  |  |  |  |
| Male | 1.08 (0.92-1.27) | 1.02 (0.87-1.20) | 1.08 (0.93-1.24) | 1.00 (0.87-1.16) |
| Female | Ref. | Ref. | Ref. | Ref. |
| Age Group |  |  |  |  |
| 13-14 | 1.08 (0.92-1.27) | 1.24 (1.05-1.47)* | 0.97 (0.84-1.12) | 1.17 (1.01-1.36)* |
| 15-17 | Ref. | Ref. | Ref. | Ref. |
| Smoking Status |  |  |  |  |
| Current Smoker | 3.10 (2.45-3.91)*** | 1.71 (1.29-2.28)*** | 4.52 (3.68-5.56)*** | 2.43 (1.89-3.12)*** |
| Former Smoker | 2.51 (2.08-3.04)*** | 1.81 (1.46-2.26)*** | 3.96 (3.35-4.68)*** | 2.60 (2.15-3.15)*** |
| Never Smoker | Ref. | Ref. | Ref. | Ref. |
| JUUL Use Status |  |  |  |  |
| Current JUUL User | 3.67 (2.97-4.54)*** | 2.58 (2.00-3.35)*** | 4.65 (3.85-5.62)*** | 2.64 (2.10-3.33)*** |
| Former JUUL User | 2.88 (2.23-3.72)*** | 2.15 (1.63-2.84)*** | 4.78 (3.87-5.91)*** | 2.91 (2.30-3.67)*** |
| Never JUUL User | Ref. | Ref. | Ref. | Ref. |

Supplementary Table F. (continued)

|  | Neither Likely Nor Unlikely | | Somewhat Likely | |
| --- | --- | --- | --- | --- |
| Predictor Variable | Unadjusted OR (95% CI) | aOR  (95% CI) | Unadjusted OR (95% CI) | aOR  (95% CI) |
| Sex |  |  |  |  |
| Male | 1.14 (1.00-1.29) | 1.07 (0.94-1.22) | 1.03 (0.94-1.14) | 1.01 (0.91-1.11) |
| Female | Ref. | Ref. | Ref. | Ref. |
| Age Group |  |  |  |  |
| 13-14 | 0.86 (0.75-0.98)* | 0.99 (0.87-1.14) | 0.95 (0.86-1.04) | 1.02 (0.93-1.13) |
| 15-17 | Ref. | Ref. | Ref. | Ref. |
| Smoking Status |  |  |  |  |
| Current Smoker | 3.78 (3.12-4.58)*** | 2.25 (1.79-2.83)*** | 1.80 (1.53-2.11)*** | 1.37 (1.13-1.65)** |
| Former Smoker | 2.88 (2.46-3.36)*** | 2.08 (1.75-2.48)*** | 1.90 (1.68-2.13)*** | 1.57 (1.38-1.79)*** |
| Never Smoker | Ref. | Ref. | Ref. | Ref. |
| JUUL Use Status |  |  |  |  |
| Current JUUL User | 3.74 (3.13-4.47)*** | 2.21 (1.78-2.75)*** | 1.96 (1.69-2.28)*** | 1.55 (1.30-1.85)*** |
| Former JUUL User | 3.26 (2.65-4.01)*** | 2.14 (1.70-2.68)*** | 2.11 (1.78-2.50)*** | 1.68 (1.40-2.02)*** |
| Never JUUL User | Ref. | Ref. | Ref. | Ref. |

*** p < 0.001; ** p < 0.010; * p < 0.050; The reference category for the dependent variable was: ‘very likely’.

**Note**: The question was: *“How likely is someone to become addicted to using a JUUL e-cigarette?”*.

**Abbreviations**: aOR = adjusted odds ratio. Unadjusted ORs were estimated using only the relevant variable as the predictor variable.

Supplementary Table G. Multinomial logistic regression model information for factors associated with perceptions of the harmfulness of using a JUUL e-cigarette compared to smoking cigarettes.

|  | JUUL Less Harmful | | JUUL More Harmful | | Don’t Know | |
| --- | --- | --- | --- | --- | --- | --- |
| Predictor Variable | Unadjusted OR (95% CI) | aOR  (95% CI) | Unadjusted OR (95% CI) | aOR  (95% CI) | Unadjusted OR (95% CI) | aOR  (95% CI) |
| Sex |  |  |  |  |  |  |
| Male | 1.09 (1.00-1.20) | 1.05 (0.95-1.15) | 1.10 (0.96-1.25) | 1.06 (0.93-1.21) | 0.93 (0.81-1.10) | 0.96 (0.83-1.10) |
| Female | Ref. | Ref. | Ref. | Ref. | Ref. | Ref. |
| Age Group |  |  |  |  |  |  |
| 13-14 | 0.93 (0.85-1.02) | 1.05 (0.96-1.16) | 1.10 (1.00-1.30) | 1.15 (1.00-1.32)* | 1.20 (1.00-1.33)* | 1.09 (0.95-1.26) |
| 15-17 | Ref. | Ref. | Ref. | Ref. | Ref. | Ref. |
| Smoking Status |  |  |  |  |  |  |
| Current Smoker | 2.21 (1.93-2.54)*** | 1.06 (0.89-1.25) | 1.97 (1.64-2.40)*** | 1.60 (1.25-2.00)*** | 0.41 (0.30-0.55)*** | 0.56 (0.39-0.79)** |
| Former Smoker | 2.27 (2.04-2.53)*** | 1.47 (1.30-1.65)*** | 1.20 (1.00-1.40) | 1.10 (0.91-1.32) | 0.65 (0.53-0.80)*** | 0.78 (0.63-1.00)* |
| Never Smoker | Ref. | Ref. | Ref. | Ref. | Ref. | Ref. |
| JUUL Use Status |  |  |  |  |  |  |
| Current JUUL User | 3.53 (3.09-4.03)*** | 3.20 (2.73-3.76)*** | 1.99 (1.65-2.41)*** | 1.60 (1.30-2.00)*** | 0.41 (0.29-0.56)*** | 0.56 (0.40-0.81)** |
| Former JUUL User | 2.97 (2.57-3.43)*** | 2.52 (2.16-2.95)*** | 1.12 (0.88-1.42) | 1.00 (0.77-1.30) | 0.48 (0.35-0.67)*** | 0.58 (0.41-0.82)** |
| Never JUUL User | Ref. | Ref. | Ref. | Ref. | Ref. | Ref. |

*** p < 0.001; ** p < 0.010; * p < 0.050; The reference category for the dependent variable was: ‘equally harmful’.

**Note**: The question was: *“Do you believe using a JUUL e-cigarette is less harmful, about the same, or more harmful than smoking cigarettes?”*.

**Abbreviations**: aOR = adjusted odds ratio. Unadjusted ORs were estimated using only the relevant variable as the predictor variable.

Supplementary Table H. Multinomial logistic regression model information for factors associated with perceptions of the addictiveness of using a JUUL e-cigarette compared to smoking cigarettes.

|  | JUUL Less Addictive | | JUUL More Addictive | | Don’t Know | |
| --- | --- | --- | --- | --- | --- | --- |
| Predictor Variable | Unadjusted OR (95% CI) | aOR  (95% CI) | Unadjusted OR (95% CI) | aOR  (95% CI) | Unadjusted OR (95% CI) | aOR  (95% CI) |
| Sex |  |  |  |  |  |  |
| Male | 1.10 (1.01-1.21)* | 1.10 (0.96-1.16) | 1.09 (1.00-1.24) | 1.06 (0.93-1.21) | 0.98 (0.84-1.13) | 0.99 (0.90-1.16) |
| Female | Ref. | Ref. | Ref. | Ref. | Ref. | Ref. |
| Age Group |  |  |  |  |  |  |
| 13-14 | 0.91 (0.83-1.00)* | 1.04 (0.94-1.14) | 1.20 (1.02-1.33)* | 1.20 (1.03-1.35)* | 1.22 (1.04-1.41)* | 1.15 (0.99-1.35) |
| 15-17 | Ref. | Ref. | Ref. | Ref. | Ref. | Ref. |
| Smoking Status |  |  |  |  |  |  |
| Current Smoker | 2.53 (2.21-3.00)*** | 1.33 (1.13-1.57)** | 1.60 (1.30-2.00)*** | 1.43 (1.14-1.80)** | 0.40 (0.28-0.60)*** | 0.60 (0.42-0.90)** |
| Former Smoker | 2.60 (2.34-3.00)*** | 1.75 (1.55-1.98)*** | 0.93 (0.78-1.10) | 0.77 (0.77-1.12) | 0.71 (0.58-0.90)** | 0.87 (0.70-1.08) |
| Never Smoker | Ref. | Ref. | Ref. | Ref. | Ref. | Ref. |
| JUUL Use Status |  |  |  |  |  |  |
| Current JUUL User | 3.43 (3.04-3.88)*** | 2.72 (2.34-3.15)*** | 1.44 (1.20-1.74)*** | 1.25 (0.99-1.60) | 0.34 (0.24-0.50)*** | 0.44 (0.30-0.70)*** |
| Former JUUL User | 3.00 (2.52-3.32)*** | 2.21 (1.90-2.60)*** | 0.92 (0.72-1.20) | 0.88 (0.70-1.14) | 0.59 (0.44-0.81)** | 0.68 (0.50-0.94)* |
| Never JUUL User | Ref. | Ref. | Ref. | Ref. | Ref. | Ref. |

*** p < 0.001; ** p < 0.010; * p < 0.050; The reference category for the dependent variable was: ‘equally addictive’.

**Note**: The question was: *“Do you believe using a JUUL e-cigarette is less addictive, about the same, or more addictive than smoking cigarettes?”*.

**Abbreviations**: aOR = adjusted odds ratio. Unadjusted ORs were estimated using only the relevant variable as the predictor variable.
